# Postoperative changes in body composition after laparoscopic and open resection of colorectal liver metastases: data from the randomized OSLO-COMET trial

**DOI:** 10.1007/s00464-025-11613-8

**Published:** 2025-02-24

**Authors:** Martin Alavi Treider, Elisa Romandini, Dena Treider Alavi, Davit Aghayan, Margrethe K. Rasmussen, Giovanni Marchegiani, Peter M. Lauritzen, Egidijus Pelanis, Bjørn Edwin, Rune Blomhoff, Åsmund Avdem Fretland

**Affiliations:** 1 Department of Gastrointestinal Surgery, Oslo University Hospital, Oslo, Norway; 2 Institute of Clinical Medicine, University of Oslo, Oslo, Norway; 3 Department of Hepato-Pancreato-Biliary Surgery, Oslo University Hospital, Oslo, Norway; 4 Department of General and Pancreatic Surgery, Verona University Hospital, Verona, Italy; 5 Department of Gastrointestinal Surgery, Hamar Hospital, Hamar, Norway; 6 Department of Nutrition, Institute of Basic Medical Sciences, University of Oslo, Oslo, Norway; 7 The Intervention Centre, Oslo University Hospital, Oslo, Norway; 8 Department of Surgery, Vestre Viken Hospital Trust, Ringerike Hospital, Hønefoss, Norway; 9 Department of Surgery N1, Yerevan State Medical University After M. Heratsi, Yerevan, Armenia; 10 Department of Hepato-Pancreato-Biliary and Liver Transplant Surgery, Padua University Hospital, Padua, Italy; 11 Department of Life Sciences and Health, Oslo Metropolitan University, Oslo, Norway; 12 Division of Radiology and Nuclear Medicine, Oslo University Hospital, Oslo, Norway; 13 Department of Clinical Service, Division of Cancer Medicine, Oslo University Hospital, Oslo, Norway

**Keywords:** Laparoscopic liver surgery, Artificial intelligence, Body composition, Colorectal liver mestastasis

## Abstract

**Background:**

Low muscle mass is negatively associated with survival in patients undergoing surgery for colorectal cancer. Current evidence is limited regarding whether the surgical approach for liver resection of colorectal metastasis impacts postoperative changes in body composition and whether preoperative body composition can impact complication rate and survival.

**Method:**

This study included patients previously included in the randomized OSLO-COMET trail where patients was allocated to laparoscopic or open liver resection for colorectal liver metastasis. CT scans 0–3 months before and 2–6 months after liver resection were segmented with the artificial intelligence-based tool BodySegAI to measure skeletal muscle mass (SM), visceral adipose tissue (VAT), and inter- and intramuscular adipose tissue (IMAT). SM, VAT and IMAT was compared between the open and laparoscopic group and as predictors for 5-year survival and postoperative complications.

**Results:**

This study included 216 patients, median age was 67, 127 (59%) were male, 91 (42%) had primary tumor in rectum and 86 (40%) had multiple liver metastasis. There was no significant difference in postoperative change in SM, VAT or IMAT between those undergoing laparoscopy or open surgery. In multivariate analysis, high preoperative IMAT was a predictor for increased risk of postoperative complications (HR (95% CI): 1.045 (CI 95%: 1.003–1.089), p = 0.034). Moreover, postoperative increase in IMAT was a negative predictor for 5-year survival (HR (95%CI):1.009 (1.003–1.016), p = 0.003).

**Conclusion:**

Postoperative change in body composition did not differ between patients randomly assigned to open or laparoscopic liver resection for colorectal metastasis. High preoperative IMAT was associated with an increased risk of postoperative complications.

Colorectal cancer (CRC) is one of the most common cancers worldwide. Overall, it ranks third in terms of incidence and second in terms of mortality [3, 4]. Moreover, the liver is the most common site of metastases from colorectal cancer. Despite the advances in oncological treatment, surgical removal of CRC liver metastases remains the main potentially curative treatment. Among those with liver-limited colorectal metastases, 0%−30% of patients have potentially resectable disease, with 5-year survival rates of 40% to 57% after liver resection [5–7]. Patients with colorectal cancer are at risk of sarcopenia (loss of muscle mass and strength) secondary to the malignancy, treatment, physical inactivity and loss of appetite [8, 9]. In a large cohort of patients with colorectal cancer, the reported rate of sarcopenia was 46%, similar to hospitalized patients > 80 years of age, and more than twice than in healthy people < 70 years [10–12]. The combination of high visceral adipose tissue and low skeletal muscle mass is associated with prolonged recovery and worsened oncological outcomes, particularly in CRC patients [13–15]. Complications to surgery remain a cause of treatment failure, especially for sarcopenic patients. The incidence of postoperative complications is higher in these patient groups, but it is still challenging to predict complications on an individual patient level [16].

Because of its availability in oncological patients, objectivity, repeatability, and accuracy, Computed Tomography (CT) is widely used in the clinic and can additionally be used to measure body composition. With errors ranging from 1 to 4%, CT is often considered a reference standard for the measurement of body composition [17, 18]. CT scans are routinely obtained in oncology patients for diagnosis, surgical planning, and follow-up. Measurements of muscle and adipose tissue from CT scans provide information about body composition, which may predict surgical complications [19–21], chemotherapy treatment toxicity [22], morbidity, and mortality after a cancer diagnosis [15, 19, 23]. BodySegAI is a newly developed software based on deep learning that automatically quantifies skeletal muscle mass (SM), subcutaneous and visceral adipose tissue (SAT and VAT), and inter- and intramuscular adipose tissue (IMAT) from routinely acquired CT scans of CRC patients. When compared to human segmentation, the BodySegAI has demonstrated promising results [24–26].

Recent studies showed reduced negative effects on muscle mass after laparoscopy compared to open surgery in CRC patients [9, 27]. Whether the laparoscopic approach reduces post-operative loss of muscle mass itself is yet unclear. A few studies have compared changes in muscle mass after laparoscopic and open gastrointestinal surgery. Most of these have been conducted on patients undergoing gastrectomy and the overall results show no difference in post-operative loss of muscle mass comparing laparoscopic and open approaches [28–30]. In CRC patients, on the other hand, laparoscopy has been reported to reduce postoperative loss of muscle mass compared to open surgery [31].

In the OSLO-COMET trial, patients were randomly assigned to open or laparoscopic resection of colorectal liver metastases [32]. In a Health-Related Quality of Life (HRQoL) analysis from OSLO-COMET, patients in the open surgery group reported reduced score for the “role physical” domain in the short-form 36 questionnaire up to four months after surgery [33]. Furthermore, the OSLO-COMET study showed significantly reduced surgical complications, less inflammation, and a shorter length of stay after laparoscopy [5]. Thus, we hypothesized that patients undergoing laparoscopic liver resection for CRC metastasis had an improved body composition post-operatively compared to patients undergoing open liver resection.

The primary aim of this study was to compare the postoperative changes in abdominal skeletal muscle mass and fat distribution in patients undergoing laparoscopic and open liver surgery. The secondary aims were to investigate whether preoperative body composition could predict postoperative complications or survival.

## Methods

This study analyzed body composition of patients previously included in the randomized OSLO-COMET trial [5]. The cohort consists of patients with CRC liver metastases resected by laparoscopic or open parenchyma-sparing liver resection from February 2012 to January 2016 at Oslo University Hospital, a tertiary referral center in Norway. The trial was approved by the Regional Committee for Medical and Health Research Ethics of Southeast Norway (“REK-Sør-Øst B 2011/1285”) and the local data protection official, and the patients had to sign a written consent to be eligible for the trial.

All patients previously included in the OSLO-COMET trial were eligible for this study. Patients with CRC liver metastases eligible for parenchyma-sparing liver resection (fewer than 3 consecutive segments) were included in the OSLO-COMET trial, including those with recurrent metastases after prior liver surgery or resectable lung or adrenal metastases. Patients did not undergo concomitant procedures during liver surgery. Exclusions included other extrahepatic metastases, planned ablation, vascular or biliary reconstruction, or simultaneous primary tumor resection. The exclusion criteria for this present study were: No retrievable preoperative CT scan within 3 months before surgery or postoperative CT scan between 2 and 6 months after surgery, patients not treated per protocol and not included in the survival analysis, CT scans with SM outside the field of view or CT scans with error in segmentation due to noise or artefacts. If more than one CT scan was available within this time range, the preoperative CT scan closest to the date of surgery and the postoperative CT scan closest to 4 months after surgery were chosen.

All CT scans in this study were conducted as part of the clinical investigation and follow-up of the patients and were analyzed retrospectively. Most of the CT scans were conducted at the patient’s local hospital. Patient demographics, oncological treatment, surgical method, Physical Status Classification System (ASA score) and Performance Status Scale (ECOG score), and postoperative complications were retrieved from the OSLO-COMET dataset. Postoperative complications were categorized with the accordion severity grading system of surgical complications (ACR). All complications ACR ≥ 2 were included in the analysis.

CT scans were processed in SECTRA PACS (Sectra Workstation IDS7 v 24.1, Sectra AB, Linköping, Sweden) Siemens syngo.via (Siemens Healthineers, Erlangen, Germany) and ITK-SNAP 4.0 (www.itksnap.org). Single slices were acquired at the mid-L3 level. Segmentation was performed by BodySegAI [26]. In case of larger errors that were suspected to influence the results, manual corrections were done using the semi-manual segmentation tool MedSeg (medseg.ai) with predefined thresholding values established by the Alberta protocol [34]. Skeletal muscle mass (SM), visceral adipose tissue (VAT), and inter- and intramuscular adipose tissue (IMAT) were segmented in pre-and postoperative CT scans. The differences between the time points were termed “Postoperative change” and calculated as post minus preoperative values. If defined as a percentage, the abovementioned difference was divided by the preoperative value and multiplied by 100.

## Statistics

Median and Interquartile Range (IQR) values were used for reporting continuous variables, while categorical variables were presented by frequencies (percentages). A two-sample Student’s t-test was applied to compare normally distributed continuous data, and the Mann–Whitney U test was applied for non-normally distribution. Two-tailed p-value < 0.05 was considered statistically significant.

To identify risk factors for postoperative complications, uni- and multivariate binary logistic regression analyses were conducted. Similarly, uni- and multivariable Cox-regression analyses were performed to identify predicators for survival. Survival follow-up started at the time of liver resection.

A set of univariate variables with expected clinical relevance was chosen, and variables associated with postoperative complications with p-values ≤ 0.2 in the univariate were subsequently included in the multivariate regression model, with significance set at p-values ≤ 0.05. This approach was also adopted in the Cox-regression analyses.

The statistical analysis was carried out using SPSS software (IBM Corp. Released 2013. IBM SPSS Statistics for Windows, version 29.0, Armonk, NY, USA: IBM corp.).

## Results

**Fig. 1:**
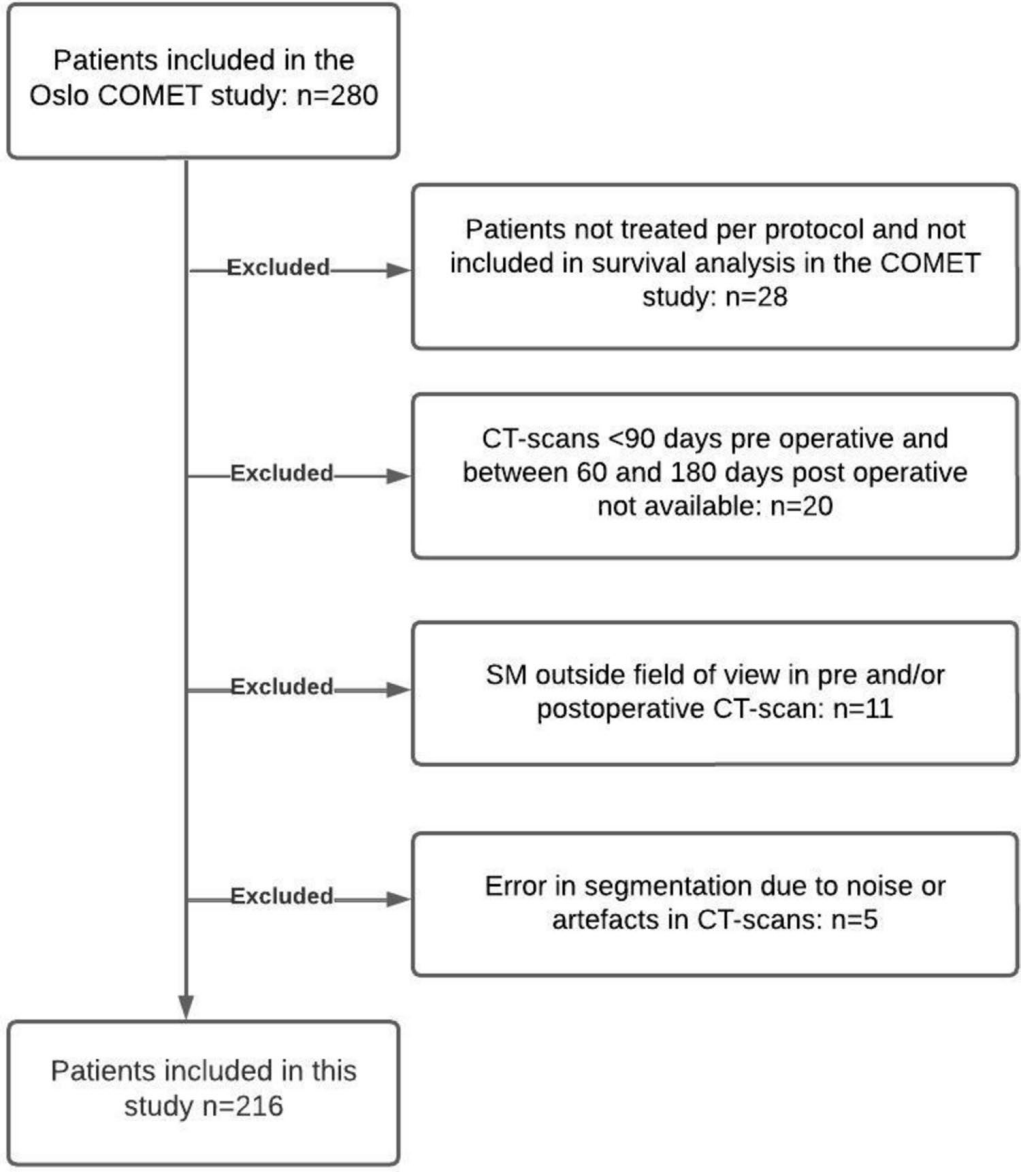
Flow diagram of patient inclusion and exclusion

Of 280 patients in the OSLO-COMET study, 216 met inclusion criteria for the current study (Fig. 1). The median age was 67 (60–74), 127 (59%) were male gender and the median BMI was 24.6 (22.3–27.4). Ninty-one (42%) had primary tumor in rectum, median tumor size was 24 (15–36) mm, 126 (58%) had synchronous metastases, 86 (40%) had multiple liver metastases, and 25 (12%) had extrahepatic metastases. Laparoscopic liver resection was performed in 103 (48%) patients, 30 had undergone a previous liver resection, 21 (10%) underwent liver surgery before primary colorectal surgery, 112 (52) had undergone neoadjuvant chemotherapy and 105 (49%) underwent adjuvant chemotherapy within 4 months after surgery. Postoperative complications occurred in 56 (25%) of the patients. There were no differences in patient characteristics between the open and laparoscopic group (Table 1). The preoperative CT scans were performed in median 49 (32–74) days before liver resection, and the postoperative CT scans in median 121 (112–134) days after liver resection. Baseline body composition data are presented in Table 2. There was no significant difference between the laparoscopic and open surgery groups with regard to change in body composition from before to after surgery (Table 3).

**Table 1:** Patients and baseline characteristics of patients in the laparoscopic and open group. Numbers are n (%) unless otherwise specified. Numbers are median (IQR)

Variable	Lap (*n* = 103)	Open (*n* = 113)	p-value
Age (years), median (IQR)	68 (61 to 75)	66 (60 to 73)	0.622
Male gender, n (%)	57 (55%)	70 (62%)	0.336
BMI (kg/m2), median (IQR)	24.6 (22.5 to 27.9)	24.3 (22.1 to 27.4)	0.535
ASA score 1 2 3 4	8 50 45 –	17 59 37 –	0.118
ECOG score 0 1 2	86 16 1	92 21 -	0.529
Primary tumor in the rectum	40 (39%)	51 (45%)	0.408
Primary tumor lymph node metastasis	71 (69%)	70 (62%)	0.318
Primary tumor T stage T3 or T4	99 (96%)	103 (91%)	0.172
Synchronous metastasis	57 (55%)	69 (62%)	0.406
Patients with multiple liver metastases	35 (34%)	51 (45%)	0.098
Extrahepatic metastasis at liver resection	15 (15%)	10 (9%)	0.208
Chemotherapy prior surgery	46 (45%)	66 (58%)	0.056
CEA level (µg/L), median (IQR)	3.7 (2.4 to 9.2)	4.2 (2 to 8)	0.742
Tumor size (mm), median (IQR)	24 (15 to 36)	25 (15 to 36)	0.954
Previous liver resection	19 (18%)	11 (10%)	0.077
Postoperative Complications Grade 2 Grade 3 Grade 4 Grade 5	22 (21%) 9 10 1 2	34 (30%) 19 8 6 1	0.144

**Table 2:** Computer tomography (CT) scan and body composition data before and after surgery. Numbers are median (IQR)

Variable	*n* = 216
Pre SM, cm^2^ median (IQR)	134.8 (104.3 to 157.6)
Pre IMAT, cm^2^ median (IQR)	9.8 (6.1 to 14.8)
Pre VAT, cm^2^ median (IQR)	118.8 (60.5 to 198.9)
Postop. change SM, cm^2^ median (IQR)	2 (−3.4 to 8.23)
Postop. change IMAT, cm^2^ median (IQR)	−9.0 (−26.2 to 35.3)
Postop. change VAT, cm^2^ median (IQR)	−5.4 (−19.6 to 11.35)

*CT* Computed tomography, *IQR* Interquartile range, *SM* Skeletal muscle mass, *IMAT* Inter- and intramuscular adipose tissue, *VAT* Visceral adipose tissue

**Table 3:** Comparison between laparoscopy and open surgery. Numbers are median (IQR)

Parameter	Laparoscopy	Open surgery	P value
	*n* = 103	*n* = 113	
Interval between preop CT and liver resection, days median (IQR)	53 (28 to 71)	48 (34 to 76)	0.810
Interval between liver resection and postop. CT, days median (IQR)	120 (111 to 137)	122 (112 to 132)	0.866
Pre SM, cm^2^ median (IQR)	130.5 (103.3 to 156.7)	135.8 (104.7 to 159)	0.760
Pre IMAT, cm^2^ median (IQR)	10.0 (6.7 to 15.4)	9.6 (5.4 to 14.1)	0.440
Pre VAT, cm^2^ median (IQR)	120.1 (53.7 to 196)	117.5 (70.9 to 201.9)	0.973
Postop. change SM, cm^2^ median (IQR)	2.3 (− 2.0 to 9.0)	1.6 (− 4.7 to 7.9)	0.296
Postop. change IMAT, cm^2^ median (IQR)	−8.70 (−26.5 to 14.2)	−9.20 (−24.2 to 9.7)	0.876
Postop. change VAT, cm^2^ median (IQR)	−4.80 (−18.2 to 10.1)	−5.50 (−21.7 to 11.9)	0.676

*CT* computed tomography, *IQR* Interquartile range, *SM* Skeletal muscle mass, *IMAT* Inter- and intramuscular adipose tissue; *VAT* Visceral adipose tissue. *NS* not significant (*p* > 0.05)

In the multivariate analysis, no pre-operative body composition factors significantly correlated with survival. However, higher preoperative IMAT was associated with an increased risk of postoperative complications. The Receiver Operating Characteristics (ROC) analysis showed an increased risk of complications when presenting with an IMAT of 9.8 cm^2^ or higher. The complication rate increased with increased preoperative IMAT. Patients with a preoperative IMAT < 10 cm^2^, 10–20 cm^2^, and > 20 cm^2^ had a complication rate of 20/112 (18%), 24/76 (32%), and 12/28 (43%), respectively. Furthermore, an increase in IMAT from before to after surgery was associated with reduced overall survival. Tumor size and Extrahepatic metastasis also predict worse survival (Table 4).

**Table 4:** Multivariate analysis of risk factors for overall survival and postoperative complications

Variable (*n* = 216)	HR (95% Cl)	p-value
Postoperative complications		
Pre IMAT	1.045 (1.003–1.089)	0.034
Bilobar tumor	2.303 (1.142–4.645)	0.020
Male gender	1.891 (0.947–3.776)	0.071
Tumor size (cm)	1.271 (1.067–1.514)	0.007
5-years overall survival		
ECOG	1.478 (0.950–2.300)	0.083
Node	1.395 (0.944–2.063)	0.095
Tumor size (cm)	1.177 (1.086–1.276)	< 0.001
Extrahepatic metastasis	1.727 (1.046–2.850)	0.033
Postoperative change IMAT	1.009 (1.003–1.016)	0.003

*HR* Hazard ratio, *IMAT* Inter- and intramuscular adipose tissue, *CI* Confidence Interval

## Discussion

This is the first study to compare changes in body composition after laparoscopic and open liver surgery. The surgical approach did not seem to impact postoperative loss of muscle mass or other changes in body composition. High preoperative intramuscular adipose tissue (IMAT) was predictive of postoperative complications, while an increase in IMAT after surgery was predictive of reduced long-term survival.

In the OSLO-COMET trial, enhanced recovery after surgery (ERAS) protocol was applied in all patients, and all patients were treated with parenchyma-sparing techniques, with relatively low complication rates and short length of stay [5]. This could explain the fact that open liver surgery did not cause more loss of muscle mass than laparoscopic surgery. However, this might be different in liver surgery with higher morbidity rates and larger surgical trauma, such as for hepatocellular carcinoma or cholangiocarcinoma [35, 36]. Similar results with no difference in postoperative change in muscle mass between open and laparoscopic surgery were reported in a study on distal gastrectomy [37]. Subtotal gastrectomy is commonly accompanied by postoperative feeding difficulties, and patients in both the laparoscopic and open groups had postoperative muscle mass loss. The postoperative muscle loss in both groups after gastrectomy makes it difficult to evaluate the isolated effect of the surgical approach on muscle loss. This study supports current evidence that postoperative change in body composition is a complex process and might not be affected by the surgical approach alone.

High preoperative IMAT was associated with an increased risk of postoperative complications. This is supported by previous studies in this field [38]. It is uncertain whether IMAT has a causal relationship with postoperative complications. However, IMAT is a marker of frailty and is associated with increased surgical risk [39]. Our study also suggests cut-off values for IMAT with regards to increased risk of postoperative complications. The cut-off value of 9.8 cm^2^ is somewhat lower than IMAT values of living kidney donors (13.4cm^2^), which is one of few healthy populations undergoing CT scans [40]. This suggests that high IMAT could be a risk factor, while lower IMAT levels compared to the healthy population might offer a protective effect against postoperative complications. Using a semi-automated tool such as Body Seg AI, this has the potential to be included in clinical practice, due to its speed, accuracy, and reproducibility [24–26]. By identifying patients at risk of developing postoperative complications preoperatively based on IMAT levels, targeted pre-habilitation may be offered to surgical patients. Diet and physical activity interventions have shown promising results in reducing IMAT levels and implementing these interventions before surgery could potentially mitigate the risk of postoperative complications [41, 42]. Furthermore, increased IMAT might be used as a frailty marker leading to either further frailty investigations or serve as information to guide the patient in a shared-decision making conversation. IMAT is also an objective measurement, meaning that it can be used to evaluate the patient’s physical status at the time of referral and thereby identify fragile patients at an earlier stage. Further studies investigating preoperative body composition with a prospective design are needed to develop this method. Moreover, preoperative predictors such as IMAT may be even more useful in populations with higher postoperative morbidity and mortality than parenchyma-sparing liver resection for colorectal metastasis.

Postoperative changes in IMAT were associated with survival; a postoperative increase in IMAT with 10 cm^2^ increased the risk of death within 5 years by 9%. The increase in intramuscular adipose tissue could indicate that patients are in a catabolic phase, most likely not due to liver resection, but due to an aggressive disease biology. This means that postoperative increase in IMAT can be one of many factors reflecting aggressive disease. However, the impact of increased IMAT alone on survival seems to be more limited. Preoperative body composition did not impact survival in this study. This is contrary to many other publications analyzing changes in body composition after GI oncological surgery such as esophagectomy [41] or surgery for CRC [8, 27, 42]. A possible explanation for this difference could be that most patients in this study had recently undergone resection of the colon or rectum, as well as chemotherapy or radiation therapy. The multiple treatments that these patients had already undergone in a short time may explain why preoperative body composition at this specific time point in the middle of a treatment chain did not create an impact on survival. An analysis of body composition at time of diagnosis might have led to different findings, but this was not the focus of the current study.

The main strength of this study is that the data are based on a randomized controlled trial and has mature survival data with a minimum follow-up of 6 years. Moreover, body composition data were retrieved from multiple CT scans from different scanners using a validated semi-automated AI-based tool, suggesting that the technique could be reproducible in other settings. The main limitation of this study was the difference in time between preoperative and postoperative CT scans due to its retrospective design. The patients had to survive until the postoperative CT scan to be included in this study and this could be a source of immortal time bias.

To conclude, postoperative change in body composition did not differ between patients randomly assigned to open or laparoscopic liver resection for colorectal metastasis. High preoperative IMAT was associated with an increased risk of postoperative complications. This study also presents cut-off values for preoperative IMAT that could serve as basis for new studies on preoperative risk factors for complications following abdominal cancer surgery.

## Abbreviations

SM: Skeletal muscle mass
SAT: Subcutaneous adipose tissue
VAT: Visceral adipose tissue
IMAT: Inter- and intramuscular adipose tissue
Diff SM: Segmented postoperative skeletal muscle mass Segmented preoperative skeletal muscle mass
Diff IMAT: Segmented postoperative inter- and intramuscular adipose tissue Segmented preoperative inter- and intramuscular adipose tissue
Diff VAT: Segmented postoperative visceral adipose tissue Segmented preoperative visceral adipose tissue
BMI: Body mass index, which divides an adult’s weight in kilograms by their height in meters squared (kg/m2)
ECOG score: Performance Status Scale, a score ranging from zero (“fully active”) through three (“capable of only limited self-care”) to five (“dead”) and has been adopted by the World Health Organization (WHO) as Performance Status Scale [1]
ASA score: Physical Status Classification System, a measure of physical status ranging from one for a normal healthy patient, through various levels of systemic disease, to six for a person who is brain-dead, required to be recorded routinely by anesthetists before any surgical procedure [2]
